# Trends, costs, and complications associated with after-hours surgery and unscheduled hospitalization in spinal surgery: a retrospective cohort study using a Japanese nationwide registry

**DOI:** 10.1302/2633-1462.58.BJO-2024-0026.R1

**Published:** 2024-08-09

**Authors:** Tomoyuki Tanaka, Masanao Sasaki, Junya Katayanagi, Akihiko Hirakawa, Kiyohide Fushimi, Toshitaka Yoshii, Tetsuya Jinno, Hiroyuki Inose

**Affiliations:** 1 Department of Orthopaedic Surgery, Dokkyo Medical University Saitama Medical Center, Koshigaya, Japan; 2 Department of Biostatistics, M&D Data Science Center, Tokyo Medical and Dental University, Tokyo, Japan; 3 Department of Health Policy and Informatics, Tokyo Medical and Dental University Graduate School of Medicine, Tokyo, Japan; 4 Department of Orthopedics, Graduate School, Tokyo Medical and Dental University, Tokyo, Japan

**Keywords:** Spine surgery, Trends, Aging, Unscheduled hospitalization, After-hours surgery, spinal surgeries, spinal diseases, spine, discectomy, spinal arthrodesis, laminoplasty, laminectomy, surgical site infections, degenerative diseases, fusion surgeries

## Abstract

**Aims:**

The escalating demand for medical resources to address spinal diseases as society ages is an issue that requires careful evaluation. However, few studies have examined trends in spinal surgery, especially unscheduled hospitalizations or surgeries performed after hours, through large databases. Our study aimed to determine national trends in the number of spine surgeries in Japan. We also aimed to identify trends in after-hours surgeries and unscheduled hospitalizations and their impact on complications and costs.

**Methods:**

We retrospectively investigated data extracted from the Diagnosis Procedure Combination database, a representative inpatient database in Japan. The data from April 2010 to March 2020 were used for this study. We included all patients who had undergone any combination of laminectomy, laminoplasty, discectomy, and/or spinal arthrodesis.

**Results:**

This investigation included 739,474 spinal surgeries and 739,215 hospitalizations in Japan. There was an average annual increase of 4.6% in the number of spinal surgeries. Scheduled hospitalizations increased by 3.7% per year while unscheduled hospitalizations increased by 11.8% per year. In-hours surgeries increased by 4.5% per year while after-hours surgeries increased by 9.9% per year. Complication rates and costs increased for both after-hours surgery and unscheduled hospitalizations, in comparison to their respective counterparts of in-hours surgery and scheduled hospitalizations.

**Conclusion:**

This study provides important insights for those interested in improving spine care in an ageing society. The swift surge in after-hours spinal surgeries and unscheduled hospitalizations highlights that the medical needs of an increasing number of patients due to an ageing society are outpacing the capacity of existing medical resources.

Cite this article: *Bone Jt Open* 2024;5(8):662–670.

## Introduction

With ageing, the spine degenerates. Therefore, the number of patients suffering from spinal degenerative diseases, such as lumbar spinal canal stenosis, cervical spondylotic myelopathy, and lumbar degenerative spondylolisthesis, increases as society ages.^1-4^ In these degenerative diseases, surgery is considered when symptoms do not improve with conservative treatment and there is interference with daily living or progressive worsening of paralysis.^5-7^ In addition, bones naturally weaken with age.^8^ Accordingly, the number of vertebral fractures increases as the spine becomes osteoporotic with ageing.^9^ In cases where osteoporotic vertebral fractures result in nonunion with persistent severe pain or neurological deficits, surgical intervention becomes necessary.^10,11^ Furthermore, even minor trauma can cause spinal cord injury in older age.^12,13^ Decompression surgery is often performed when paralysis persists after an acute spinal cord injury and spinal cord compression is present.^14^ Therefore, the number of spine surgeries is increasing in ageing countries,^15-17^ and the number of spine surgeries is expected to further increase worldwide as the population ages.^18,19^ In particular, there is concern that the growing use of posterior spinal instrumentation in older patients will pose challenges for healthcare systems around the world.^18^ Spinal disease is thus one of the most prevalent, life-disrupting, and costly conditions globally, imposing a significant burden on healthcare systems, with further burdens expected in the future.^20^

Typically, patients with spinal disease are seen on an outpatient basis and put on an operation waiting list. Because of comorbidities, especially in elderly patients, spinal surgery is usually scheduled after appropriate preoperative evaluation and treatment of comorbidities such as respiratory function, cardiac function, and glucose tolerance.^21-23^ However, worsening of paralysis, sudden onset of severe pain, or bladder-rectal problems may require unscheduled hospitalization or after-hours surgery. In addition, in cases of trauma, unscheduled hospitalization or emergency surgery is often unavoidable. Notably, after-hours procedures are associated with poor outcomes in various medical conditions.^24-26^ Furthermore, after-hours surgeries and unscheduled hospitalizations strain hospital resources, impeding efficient management.

The escalating demand for medical resources to address spinal diseases as society ages is an issue that requires careful evaluation. It is important to provide data that will serve as a basis for considering the appropriate allocation of medical resources for spine surgery in an ageing society. However, few studies have examined trends in spinal surgery, especially unscheduled hospitalizations or surgeries performed after hours, through large databases. Furthermore, there is limited knowledge about the impact of unscheduled hospitalizations and after-hours surgeries on costs and complications in spine surgery. Our study aimed to determine national trends in the number of spine surgeries in Japan, one of the most aged countries in the world, where about one-third of the population is aged over 65 years.^27^ We also aimed to identify trends in after-hours surgeries and unscheduled hospitalizations, and their impact on complications and costs.

## Methods

### Data source

This is a retrospective analysis of prospectively collected data obtained from a rigorously maintained national registry, the Diagnosis Procedure Combination (DPC) database.^28,29^ The database covers hospitals in all regions of Japan, and the number of beds included in the database accounted for 56.7% of the total number of beds in Japan, according to a survey conducted in fiscal year (FY) 2021. The DPC study group, which receives funding from the Japanese Ministry of Health, Labor, and Welfare,^30^ extracted anonymized DPC data from the DPC hospitals to create a secondary database, extracting datasets for specific purposes and providing them to researchers. The DPC database is employed by researchers to detect, monitor, and examine nationwide patterns in healthcare use, accessibility, quality, results, and expenses. This database encompasses a variety of information, including patient age and sex, diagnoses, and post-admission complications documented using the International Classification of Diseases, Tenth Revision (ICD-10)^31^ codes and text data in Japanese.

Due to the anonymized nature of the data, the necessity of obtaining informed consent was waived. Approval for the study was obtained from the Dokkyo Medical University Saitama Medical Center Institutional Review Board (23069).

### Patient sample

Individuals who underwent surgical intervention for spinal conditions in the cervical, thoracic, and lumbar spine between 2010 and 2019 were considered. The years 2020 and 2021 were excluded from the analysis due to the substantial influence of the COVID-19 pandemic on the operations of healthcare systems.

We included all patients who had any combination of laminectomy, laminoplasty, discectomy, and/or spinal arthrodesis. The following variables were abstracted from the DPC database: age, sex, primary diagnosis, cost, and postoperative adverse events. The primary diagnoses included spinal canal stenosis (M480), spondylosis (M47), disc herniation (M50, M51), spondylolisthesis (M430, M431), and vertebral fracture (S12, S22, S32, T08). Hospitalization costs were calculated based on the DPC classification system that determines a fixed daily cost for the combination of the patient’s illness, symptoms, and the treatment required. In addition, for operations, rehabilitation and medical fees are calculated for each medical procedure using the ‘pay-as-you-go method’, which is added to the fixed costs.

### Outcome measures

Data collected included the number of scheduled and unscheduled hospitalizations per year and the total number of spinal surgeries per year. As the database is only able to provide inpatient information, outpatient and day surgeries were not included. After-hours surgery was determined by the presence of an additional code indicating surgery performed outside regular hours. The distinction between scheduled and unscheduled hospitalization was determined by codes in the DPC database (scheduled hospitalization: 100; unscheduled hospitalization: 200, or 301 to 310). In the DPC database, the date of admission to the medical institution is determined in advance, and if the patient is admitted on that date it is entered as a scheduled hospitalization; otherwise, it is entered as an unscheduled hospitalization.

Postoperative adverse events included surgical site infection (T793, T814), sepsis (A40, A41), pulmonary embolism (I26), respiratory complications (pneumonia (J12 to J18), postprocedural respiratory disorders (J95), and respiratory failure (J96)), cardiac events (acute coronary events (I21 to I24) or heart failure (I50)), stroke (cerebral infarction or hemorrhage (I60 to I64)), spinal fluid leakage (G960, G961), haematoma (S064, S241, S341, T093), and meningitis (G001 to G003, G008 to G009, G039, A390, A392).

### Statistical analysis

We performed univariate regression analysis to evaluate the trends of the number of surgeries and hospitalizations and costs. We also performed multivariate regression analysis to compare the trend of the number of hospitalizations between scheduled and unscheduled hospitalization, and that of the number of surgeries between in hours and after hours. All outcomes were natural log-transformed before analysis. The exponentiated coefficients with 95% CIs were estimated by the analysis model which included year, scheduled/unscheduled hospitalizations (or in-hours/after-hours surgeries), and the interaction between year and scheduled/unscheduled hospitalizations (or in-hours/after-hours surgeries). Additionally, we plotted the distributions of the age of patients who underwent the surgery by year, and the ordinal outcome of the age group was analyzed by the proportional odds model.

Furthermore, we evaluated the associations between scheduled/unscheduled hospitalizations and the rate of complications. For each complication, the chi-squared test was performed. The same analysis was done for the associations between in-hours/after-hours surgeries and the rate of complications. For the detailed study of costs and complications, we used 2018 data to exclude the potential effect on the number of surgeries in 2019 due to lockdowns against COVID-19. This approach was taken because the DPC database counts 1 April to 31 March of the following year as a fiscal year. The threshold for significance was a p-value < 0.05. All analysis were performed by SAS v. 9.4 (SAS Institute, USA).

## Results

### Number of spinal surgeries

**Fig. 1:**
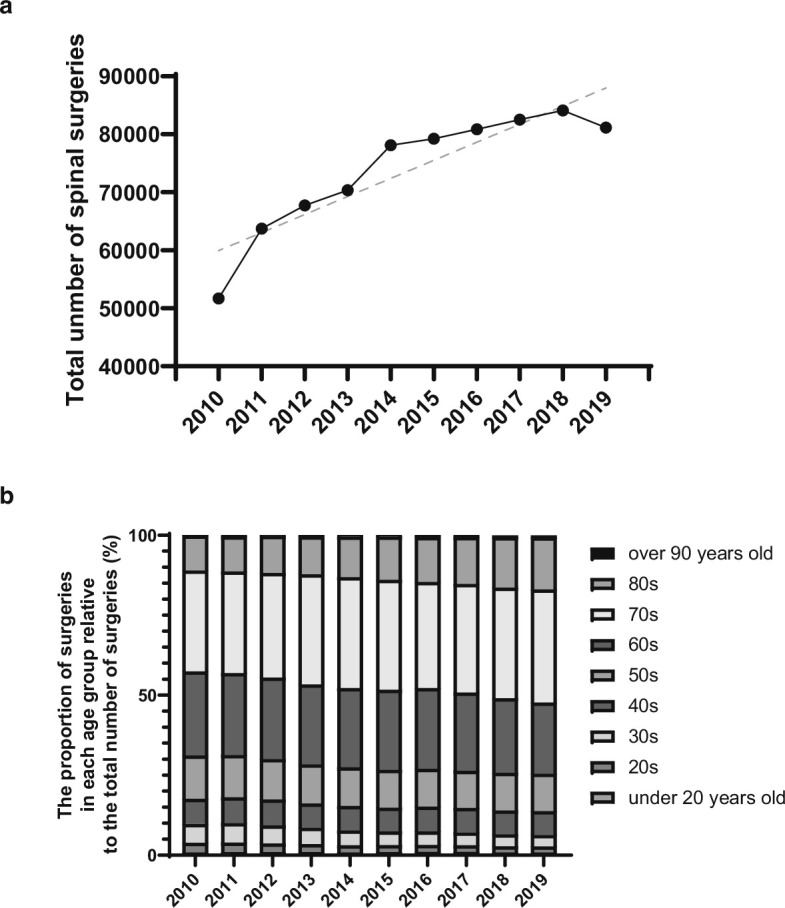
a) Scatter plots with lines demonstrating the total number of spinal surgeries in Japan from 2010 to 2019. The grey line indicates trend line. b) Trends in the age distribution of patients who underwent spine surgery in Japan from 2010 to 2019.

The investigation included 739,474 spinal surgeries conducted in Japan from 2010 to 2019 (Table I). There was an average annual increase of 4.6% in the number of spinal surgeries, with an increase rate of 1.046 (95% CI 1.031 to 1.061; p < 0.001) (Figure 1a). To provide context, the surgical interventions for spinal disease in Japan rose from 51,708 in 2010 to 81,150 in 2019 (Figure 1a). The total cost of spinal surgery in Japan increased by an average of 3.9% per year, with an increase rate of 1.039 (95% CI 1.022 to 1.057; p < 0.001) (Table I). The mean age of patients increased from 63.6 years (SD 15.4) in 2010 to 66.6 years (SD 14.9) in 2019 (mean difference 3.0, 95% CI 2.8 to 3.2; p < 0.001). The distribution of patient percentages revealed a gradual ageing trend among individuals eligible for spine surgery in Japan from 2010 to 2019 (Figure 1b) (odds ratio (OR) 1.043, 95% CI 1.041 to 1.044; p < 0.001). Of note, the percentage of patients aged 70 years and older had increased from 42.8% in 2010 to 52.3% in 2019.

**Table I.** Yearly surgical trends in Japan from 2010 to 2019.

Year	Total surgeries, n	Total costs, ¥	After-hour surgeries, n	Total hospitalizations, n	Unscheduled hospitalizations, n
2010	51,708	91,401,340,510	485	51,647	4,004
2011	63,728	103,796,840,980	612	63,687	5,335
2012	67,733	82,036,945,942	741	67,729	8,923
2013	70,348	87,616,370,162	799	70,342	9,398
2014	78,119	100,308,802,306	943	78,067	10,565
2015	79,230	10,8850,882,190	1,096	79,202	11,430
2016	80,862	116,043,081,143	1,117	80,839	12,383
2017	82,511	119,708,791,615	1,151	82,491	12,441
2018	84,085	122,424,851,405	1,127	84,069	12,229
2019	81,150	119,551,376,223	1,164	81,142	12,253
p-value*	< 0.001	< 0.001	< 0.001	< 0.001	< 0.001

* p < 0.05.

### Scheduled versus unscheduled hospitalizations

**Fig. 2:**
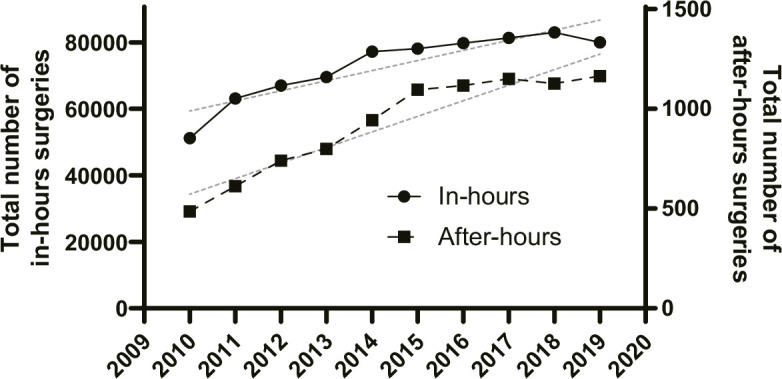
Scatter plots with lines demonstrating the total number of scheduled hospitalizations and unscheduled hospitalizations in Japan from 2010 to 2019. The grey lines indicate the respective trend lines.

This study conducted a comprehensive analysis of 739,215 hospitalizations related to spinal disease spanning the years 2010 to 2019. Among these, 640,254 hospitalizations (86.6%) were scheduled, while 98,961 (13.4%) were unscheduled. Scheduled hospitalizations showed a mean annual increase of 3.7%, as denoted by an increase rate of 1.037 (95% CI 1.006 to 1.069; p = 0.020) (Figure 2). Conversely, the number of unscheduled hospitalizations exhibited a faster annual growth rate of 11.8%, with an increase rate of 1.118 (95% CI 1.084 to 1.153; p < 0.001). Thus, unscheduled hospitalizations increased 1.078 times per year (95% CI 1.033 to 1.126; p < 0.001) compared to scheduled hospitalizations. Regarding the type of surgery, the proportion of fusion surgeries was slightly higher in the unscheduled hospitalization group compared with the scheduled hospitalization group (80.9% vs 79.7%; p < 0.001). Regarding complication rates, there was no significant difference in the incidence of haematoma or spinal fluid leakage, but surgical site infection, cardiovascular complications, sepsis, cerebrovascular complications, and urological complications were significantly higher in patients admitted unscheduled (all p < 0.001) (Table II). According to FY 2018 data, the average cost of a scheduled hospitalization was ¥1,348,242, while the average cost of an unscheduled hospitalization was ¥2,087,555 (mean difference ¥739,314, 95% CI 712,160 to 766,468; p < 0.001) (Table II).

**Table II.** Complications and costs associated with unscheduled hospitalizations.

Variable	Total (n = 84,069)	Scheduled hospitalization (n = 71,840)	Unscheduled hospitalization (n = 12,229)	p-value
**Surgical type, n (%)**				< 0.001†
Decompression surgery	16,941 (20.2)	14,606 (20.3)	2,335 (19.1)	
Fusion surgery	67,128 (79.8)	57,235 (79.7)	9,893 (80.9)	
**Postoperative complications, n (%)**				
Surgical site infection	1,559 (1.9)	1,282 (1.8)	277 (2.3)	< 0.001†
Cardiac events	621 (0.7)	462 (0.6)	159 (1.3)	< 0.001†
Respiratory complications	426 (0.5)	212 (0.3)	214 (1.8)	< 0.001†
Sepsis	144 (0.2)	51 (0.1)	93 (0.8)	< 0.001†
Pulmonary embolism	124 (0.1)	83 (0.1)	41 (0.3)	< 0.001†
Stroke	263 (0.3)	174 (0.2)	89 (0.7)	< 0.001†
Renal failure	228 (0.3)	171 (0.2)	57 (0.5)	< 0.001†
Urinary tract infection	595 (0.7)	309 (0.4)	286 (2.3)	< 0.001^†^
Haematoma	243 (0.3)	204 (0.3)	39 (0.3)	0.505
Spinal fluid leakage	240 (0.3)	206 (0.3)	34 (0.3)	0.868
Meningitis	162 (0.2)	123 (0.2)	39 (0.3)	< 0.001†
Mean cost,￥(SD)	1,455,965 (1,197,329)	1,348,242 (1,110,898)	2,087,555 (1,461,789)	< 0.001†

* Chi-squared test.

† p < 0.05

### In-hours versus after-hours surgeries

**Fig. 3:**
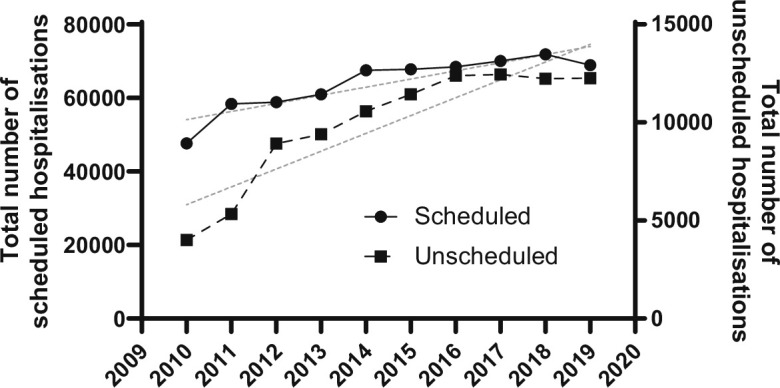
Scatter plots with lines demonstrating the total number of in-hours surgeries and after-hours surgeries in Japan from 2010 to 2019. The grey lines indicate the respective trend lines.

Surgical interventions conducted during regular working hours exhibited an average annual increase of 4.5%, as indicated by an increase rate of 1.045 (95% CI 1.025 to 1.066; p < 0.001). In contrast, surgical interventions performed after hours experienced more rapid growth, averaging 9.9% per year, with an increase rate of 1.099 (95% CI 1.077 to 1.120; p < 0.001) (Figure 3). Thus, after-hours surgeries increased 1.051 times per year (95% CI 1.022 to 1.080; p < 0.001) compared to in-hours surgeries. Regarding the type of surgery, the proportion of fusion surgeries was higher in the after-hours surgery group compared with the in-hours surgery group (94.3% vs 79.7%; p < 0.001). Regarding complication rates, while there was no significant difference in the incidence of stroke, haematoma, or spinal fluid leakage, surgical site infection, cardiac events, sepsis, pulmonary embolism, and urological complications were significantly higher in after-hours surgery (all p < 0.001, respectively) (Table III). According to FY 2018 data, the average cost of an in-hours surgery was ¥1,434,536, while the average cost of an after-hours surgery was ¥3,033,362 (mean difference ¥1,598,825, 95% CI 1,494,464 to 1,703,187; p < 0.001) (Table III).

**Table III.** Complications and costs associated with after-hours surgeries.

Variable	Total (n = 84,085)	In-hours surgery (n = 82,958)	After-hours surgery (n = 1,127)	p-value*
**Surgical type, n (%)**				< 0.001†
Decompression surgery	16,941 (20.1)	16,877 (20.3)	64 (5.7)	
Fusion surgery	67,144 (79.9)	66,081 (79.7)	1,063 (94.3)	
**Postoperative complications, n (%)**				
Surgical site infection	1,559 (1.9)	1510 (1.8)	49 (4.3)	< 0.001†
Cardiac events	621 (0.7)	601 (0.7)	20 (1.8)	< 0.001†
Respiratory complications	427 (0.5)	389 (0.5)	38 (3.4)	< 0.001†
Sepsis	144 (0.2)	126 (0.2)	18 (1.6)	< 0.001†
Pulmonary embolism	124 (0.1)	117 (0.1)	7 (0.6)	< 0.001^*^
Stroke	264 (0.3)	257 (0.3)	7 (0.6)	0.064
Renal failure	228 (0.3)	218 (0.3)	10 (0.9)	< 0.001†
Urinary tract infection	595 (0.7)	555 (0.7)	40 (3.5)	< 0.001†
Haematoma	243 (0.3)	240 (0.3)	3 (0.3)	0.886
Spinal fluid leakage	240(0.3)	234 (0.3)	6 (0.5)	0.118
Meningitis	162 (0.2)	154 (0.2)	8 (0.7)	< 0.001†
Mean cost,￥(SD)	1,455,965 (1,197,329)	1,434,536 (1,172,939)	3,033,362 (1,780,397)	< 0.001†

* Chi-squared test.

† p < 0.05.

Lastly, we investigated whether after-hours surgeries have a higher incidence of complications compared to in-hours surgeries among unscheduled hospitalization patients. Regarding complication rates, surgical site infection, respiratory complications, sepsis, and urological complications were significantly higher in after-hours surgery (all p < 0.001). According to FY 2018 data, the average cost of an in-hours surgery was ¥1,997,776, while the average cost of an after-hours surgery was ¥3,096,789 (mean difference ¥1,099,014, 95% CI 1,006,436 to 1,191,592; p < 0.001) (Table IV).

**Table IV.** Complications and costs associated with after-hours surgeries among unscheduled hospitalizations.

Variable	Total unscheduled hospitalizations (n = 12,229)	In-hours surgery (n = 11,230)	After-hours surgery (n = 999)	p-value*
**Postoperative complications, n (%)**				
Surgical site infection	277 (2.3)	241 (2.1)	36 (3.6)	< 0.001†
Cardiac events	159 (1.3)	144 (1.3)	15 (1.5)	0.558
Respiratory complications	214 (1.7)	176 (1.6)	38 (3.8)	< 0.001†
Sepsis	93 (0.8)	79 (0.7)	14 (1.4)	0.0150†
Pulmonary embolism	41 (0.3)	35 (0.3)	6 (0.6)	0.130
Stroke	89 (0.7)	84 (0.7)	5 (0.5)	0.378
Renal failure	57 (0.5)	49 (0.4)	8 (0.8)	0.105
Urinary tract infection	286 (2.3)	247 (2.2)	39 (3.9)	< 0.001†
Haematoma	39 (0.3)	38 (0.3)	1 (0.1)	0.201
Spinal fluid leakage	34 (0.3)	30 (0.3)	4 (0.4)	0.443
Meningitis	39 (0.3)	33 (0.3)	6 (0.6)	0.099
Mean cost, ￥(SD)	2,087,555 (1,461,789)	1,997,776 (1,396,084)	3,096,789 (1,772,419)	< 0.001†

* Chi-squared test.

† p < 0.05.

## Discussion

With a meticulous examination of ten years of data, encompassing 739,474 spinal surgeries and 739,215 hospitalizations, the research delves into the surgical trends associated with spinal disease. The results of this study revealed that the number and total cost of spine surgeries has increased over the years, as has the number of after-hours surgeries. This study also found that after-hours surgeries were more costly and had more complications than surgeries conducted during regular hours. Furthermore, the number of unscheduled hospitalizations has also increased, with unscheduled hospitalizations incurring increased costs and complications compared to scheduled hospitalizations.

In this study, there was an increase in the total number of spinal surgeries by 1.6 times over ten years. Additionally, the total costs associated with spine surgery have increased over time, experiencing a 1.31-fold rise from 2010 to 2019. A regional database study in Japan reported that the number of surgeries increased 1.9-fold over the 12 years from 2004 to 2015.^15^ Although the years covered by the study are slightly different, the trend in the increase in surgeries is comparable to our findings. The distribution of patient percentages showed that the distribution of patients eligible for spine surgery in Japan gradually aged from 2010 to 2019. The rise in surgical demand at a rate of 4.6% annually is probably due to the growing number of elderly individuals in Japan. Therefore, our data reveal that the increase in spinal surgeries and the associated cost increases are one of the factors that put pressure on healthcare costs in an ageing society. Other nations have also demonstrated a heightened need for spinal surgery. A study using the Finnish national database found that lumbar decompression surgeries increased 2.3-fold and lumbar fusion surgeries increased 3.3-fold between 1997 and 2018.^32^ A study using the Nationwide Inpatient Sample database in the USA found that lumbar fusion for lumbar isthmic spondylolisthesis increased 4.33-fold between 1998 and 2011.^16^ However, our report is crucial from a socioeconomic perspective as few studies have used national databases to examine comprehensive numbers and cost trends in spine surgery.

We found that unscheduled hospitalizations were increasing more rapidly than scheduled hospitalizations. An unscheduled hospitalization could mean that symptoms suddenly appeared, or that the patient was scheduled to be admitted but experienced worsening symptoms before the scheduled hospitalization date. To date, the clinical and economic implications of managing patients requiring spinal surgery with unscheduled hospitalizations have not been thoroughly examined. An important aspect is that prolonged symptoms are a risk factor for poor improvement in spine surgery,^33^ and shorter disease duration in spinal degenerative diseases implies better surgical outcomes.^34^ Our study found that patients with unscheduled hospitalizations had higher costs and complication rates than those with scheduled hospitalizations. Therefore, efforts should be made to reduce the waiting time for appointments with a spine specialist. In addition, efforts should also be made to shorten surgical waiting times for spinal degenerative diseases to reduce unscheduled hospitalizations. This will require increasing the number of spine surgeons and the proper distribution of medical resources, including the appropriate assignment of anaesthesiologists and nurses and the efficient use of operating theatres and beds. Further cost-effectiveness analysis is also necessary in considering policies for distributing medical resources for the increasing number of spine surgeries.

The fact that a spinal surgery was performed after hours means either that the patient was seen at the hospital after hours and the emergency surgery was performed directly, or that the patient was seen in hours but the surgery could not be performed in time and had to be performed after hours. We observed a more pronounced growth in after-hours surgeries than during regular hours. Similar to our research results, a retrospective study found that the percentage of patients requiring emergency surgery for degenerative spinal conditions increased from 22.6% in 2006 to 34.8% in 2019.^35^ These results suggest that rates of after-hours and emergency spine surgeries have been increasing over recent years. Additionally, our investigation revealed that patients undergoing surgeries after hours experienced elevated costs and complication rates in comparison to those undergoing procedures during regular hours. Although costs naturally increase with more complications, another reason for the increased cost of after-hours surgeries is the higher proportion of fusion surgeries performed compared to the in-hours surgery group. A retrospective study found that non-elective spine surgery performed after hours was independently associated with increased risk of perioperative adverse events, length of stay, and mortality.^36^ Interestingly, however, in another retrospective study, after-hours surgery was not associated with 30-day readmission or mortality in emergency spine surgery.^37^ In many medical fields, weekend care has been reported to have poorer outcomes than during regular weekday hours.^24,25,38^ Interestingly, in a retrospective nested-cohort study, hospitalization to the intensive care unit after hours following elective surgery was associated with increased complications and length of hospital stay compared to hospitalization to the intensive care unit in hours.^39^ While the detailed reasons for the increased number and cost of complications associated with after-hours surgery in this study are unknown, they may be related not only to inadequate preoperative assessment and management of comorbidities, but also to insufficient postoperative care after hours. The increase in the number of spine surgeries, especially after-hours surgeries, necessitates an increase in the number of spine surgeons. However, a study in Japan has reported that the number of spine surgeons has not increased as much as the number of spine surgeries.^40^ As the burden on spine surgeons increases with the ageing of society, measures will need to be taken to train and increase the number of spine surgeons with advanced expertise and skills.

The main strength of this study lies in its use of the DPC database, which covers approximately 60% of all hospital beds in Japan. Using a large, nationally representative database, we could assess the magnitude of perioperative risk and cost analysis for spine surgeries performed after hours and spine surgeries with unscheduled hospitalizations, which was not possible in previous studies.

This study has limitations inherent to administrative database research. First, the DPC database does not provide important clinical data such as the degree of pain, neurological findings, and degree of impairment to activities of daily living. Second, the possibility that a diagnosis or procedure may be entered incorrectly cannot be ruled out. Nonetheless, using large sample sizes would reduce the likelihood of this risk. Finally, the DPC database lacks post-discharge information, potentially leading to an under-representation of complications such as surgical site infections. Despite these limitations, our study contains important information for clinical practitioners and health policymakers, and provides a basis for future research. In future studies, it would be interesting to conduct similar trend analyses in countries other than Japan and other surgical specialities to assess the impact of demographic characteristics, socioeconomic status, and health insurance systems on after-hours surgery and unscheduled hospitalizations.

This study provides important insights for those interested in improving spine care in an ageing society. The results of the study revealed that the number of spine surgeries has increased over the years, especially after-hours surgeries. Furthermore, unscheduled hospitalizations have also increased, incurring increased costs and complications compared to scheduled hospitalizations. This study specifically highlights the importance of raising awareness among spine care providers and health policymakers about the increase in unscheduled hospitalizations and after-hours spinal surgeries that accompany an ageing society, and the need to reduce such occurrences.

**Take home message**

- This study used a large database to investigate trends in spine surgery in Japan from 2010 to 2019, a country with a rapidly ageing population.

- The results of this study demonstrated not only an increase over time in the number of spine surgeries, including after-hours surgeries and unscheduled hospitalizations, but also revealed that these after-hours surgeries and unscheduled hospitalizations were associated with higher complication rates and costs compared to their in-hours and scheduled counterparts, respectively.

- This trend highlights the need for improved resource management to address the escalating demands of an ageing society.

## Data Availability

The datasets generated and analyzed in the current study are not publicly available due to data protection regulations. Access to data is limited to the researchers who have obtained permission for data processing. Further inquiries can be made to the corresponding author.
